# The impact of liposomal bupivacaine erector spinae plane block on postoperative analgesia and early functional recovery in patients undergoing thoracoscopic lung cancer surgery

**DOI:** 10.3389/fonc.2026.1734623

**Published:** 2026-03-16

**Authors:** Lubin Huang, Kejing Huang, Hong Ning, Chenghai Wang

**Affiliations:** 1 Department of Anesthesiology, Central Hospital Affiliated to Shandong First Medical University, Jinan, Shandong, China; 2 Operating Room, The Fourth People’s Hospital of Jinan, Jinan, Shandong, China; 3 Operating Room, Central Hospital Affiliated to Shandong First Medical University, Jinan, Shandong, China; 4 Department of Anesthesiology, Yantaishan Hospital, Yantai, Shandong, China

**Keywords:** erector spinae plane block, functional recovery, liposomal bupivacaine, postoperative analgesia, thoracoscopic lung cancer surgery

## Abstract

**Objectives:**

This study aimed to evaluate the impact of liposomal bupivacaine erector spinae plane block (ESPB) on postoperative analgesia and early functional recovery in patients undergoing thoracoscopic lung cancer surgery.

**Methods:**

A retrospective analysis was conducted on 246 patients who underwent video-assisted thoracoscopic surgery (VATS) for lung cancer from January 2021 to December 2023. Patients were divided into two groups based on the type of local anesthetic used for ESPB: bupivacaine (n=115) and liposomal bupivacaine (n=131). Postoperative pain levels, early functional recovery, opioid consumption, pulmonary function indices, perioperative adverse events, and other postoperative-related outcomes were assessed.

**Results:**

Patients receiving liposomal bupivacaine reported lower pain scores at rest during the first 48 hours postoperatively compared to those receiving conventional bupivacaine. The liposomal bupivacaine group also demonstrated higher Quality of Recovery-15 (QoR-15) scores, reduced postoperative sufentanil consumption, and improved pulmonary function indices (FEV1 and FVC) throughout the observation period. Additionally, the time to first ambulation was shorter, and the time to the first request for analgesia was longer in the liposomal bupivacaine group (all P<0.05). No significant differences were observed in perioperative adverse events between the groups (P>0.05).

**Conclusions:**

Liposomal bupivacaine ESPB is associated with improved postoperative analgesia and earlier functional recovery in patients undergoing VATS for lung cancer. These observed benefits may be attributed to the prolonged release profile of bupivacaine, which correlates with sustained pain relief and reduced opioid requirements. This technique holds promise for optimizing postoperative care and enhancing patient recovery following thoracic surgery.

## 1 Introduction

Thoracoscopic lung cancer surgery, also known as video-assisted thoracoscopic surgery (VATS), has become a standard approach for the diagnosis and treatment of various thoracic malignancies. This minimally invasive technique offers several advantages over traditional open thoracotomy, including reduced postoperative pain, shorter hospital stays, and faster recovery times (1, 2). Despite these benefits, postoperative pain remains a significant concern for patients undergoing VATS (3). Effective management of this pain is crucial not only for enhancing patient comfort but also for facilitating early mobilization and overall recovery. Traditional analgesic methods, such as systemic opioids, often fail to provide adequate pain relief without causing significant side effects, including nausea, vomiting, respiratory depression, and increased risk of opioid dependence (4). Consequently, there is a growing interest in regional anesthesia techniques that can offer more targeted and sustained pain relief with fewer systemic effects.

Regional anesthesia techniques, particularly those involving local anesthetics, have shown promise in improving postoperative pain control. The erector spinae plane block (ESPB) is one such technique that has gained attention in recent years. ESPB involves injecting a local anesthetic into the fascial plane deep to the erector spinae muscle, targeting multiple spinal nerves that contribute to thoracic and abdominal wall innervation. Traditional bupivacaine has been widely used in ESPB, but its duration of action is limited (4, 5). Liposomal bupivacaine, which encapsulates bupivacaine within liposomes, extends the duration of analgesia by providing a slow, sustained release of the drug, which could be particularly beneficial in thoracic surgeries (6).

Pain management following thoracic surgery is closely linked to pulmonary function and early mobilization. Postoperative pain can restrict deep breathing and coughing, leading to atelectasis, pneumonia, and other respiratory complications. Effective pain control through regional anesthesia techniques like ESPB may mitigate these risks by enabling patients to breathe more deeply and effectively clear secretions. Improved pain management can facilitate earlier ambulation, which is critical for preventing thromboembolic events and accelerating overall recovery (7–9).

In addition to pain management, early functional recovery is another key aspect of postoperative care. Enhanced recovery after surgery (ERAS) protocols emphasize the importance of minimizing physiological stress and promoting rapid recovery. Effective pain control is a cornerstone of ERAS, as it enables patients to engage more actively in rehabilitation exercises and daily activities (10–12). The potential for liposomal bupivacaine ESPB to achieve these goals makes it an attractive option for optimizing postoperative care in thoracic surgery.

As such, optimizing postoperative pain management and facilitating early functional recovery are critical for enhancing patient outcomes following thoracoscopic lung cancer surgery. The use of liposomal bupivacaine in ESPB represents a promising approach to achieving these goals. This technique has been associated with prolonged and targeted pain relief, which correlates with reduced reliance on systemic opioids, better pulmonary function, and earlier mobilization in some studies. These potential benefits underscore the need for further research to validate the efficacy and safety of liposomal bupivacaine ESPB in thoracic surgery, ultimately guiding its integration into clinical practice. The present study aimed to evaluate the impact of liposomal bupivacaine ESPB on postoperative analgesia and early functional recovery in patients undergoing thoracoscopic lung cancer surgery.

## 2 Materials and methods

### 2.1 Study design and patients

**Figure 1:**
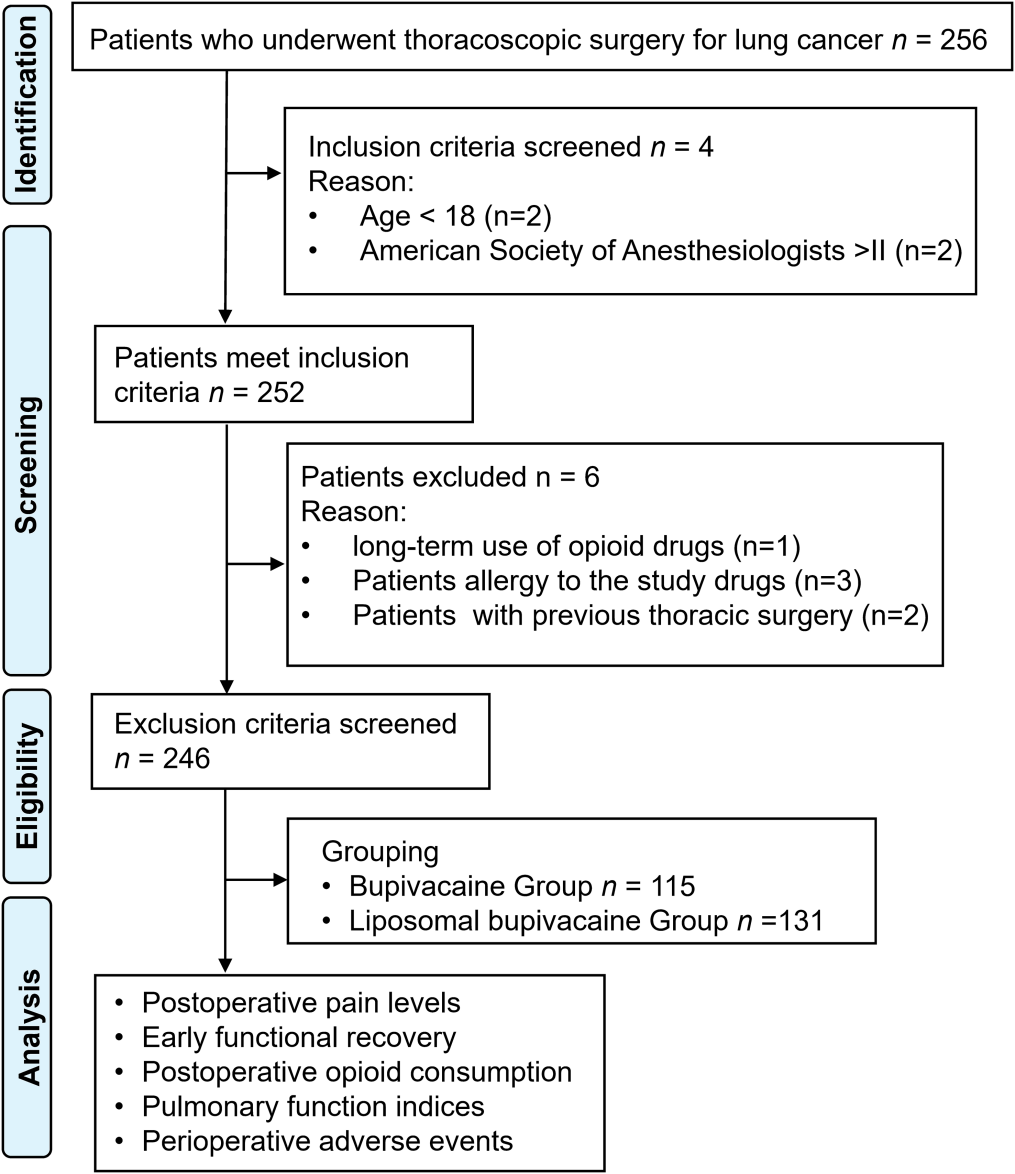
Flow diagram of patient selection.

This study conducted a retrospective analysis of 246 patients who underwent thoracoscopic surgery for lung cancer at our hospital from January 2021 to December 2023. Inclusion criteria comprised meeting the diagnostic criteria for lung cancer (13), being aged between 18 and 75 years, American Society of Anesthesiologists (ASA) (14) physical status I-II, and undergoing VATS. Exclusion criteria included long-term use of opioid drugs, allergy to the study drugs, presence of severe chronic renal or hepatic insufficiency, previous thoracic surgery, history of neuropathic pain, puncture site infection, coagulation dysfunction, and incomplete medical records (**Figure 1**). According to the different drugs used for erector spinae plane block (ESPB) during the operation, the 246 patients were defined into the bupivacaine group (n=115) and the liposomal bupivacaine group (n=131). This study was reviewed and approved by the Medical Ethics Committee of the Yantaishan Hospital (Protocol number: 2023099). Given that this is a retrospective analysis and all diagnostic and therapeutic measures were part of routine clinical practice without additional medical risk to the patients, the Ethics Committee waived the requirement for informed consent.

The choice between liposomal bupivacaine and conventional bupivacaine for ESPB was based on the chronological adoption of the newer formulation in our clinical practice. During the early phase of the study period (January 2021 to mid-2022), conventional bupivacaine was routinely used for ESPB in VATS procedures. Following institutional experience and emerging evidence supporting its prolonged analgesic effect, liposomal bupivacaine was introduced and gradually adopted as a standard option from mid-2022 onward. Therefore, the allocation was primarily time-based rather than patient-specific, reflecting a natural transition in clinical practice. All patients received the standard-of-care ESPB as per the prevailing protocol at the time of their surgery.

### 2.2 Anesthetic and surgical procedure

After the patients entered the operating room, routine monitoring included electrocardiogram (ECG), non-invasive blood pressure, pulse oxygen saturation, body temperature, end-tidal CO2, and bispectral index (BIS). Anesthesia induction was performed using propofol 2.5 mg/kg (Approval No. H20123138, Jiangsu Nhwa Pharmaceutical Co., Ltd., Jiangsu Province, China), sufentanil 0.3 μg/kg (Approval No. H20054172, Yichang Humanwell Pharmaceutical Co., Ltd., Hubei Province, China), and cisatracurium 0.2 mg/kg (Approval No. H20133373, Shanghai Dongying Pharmaceutical Co., Ltd., Jiangsu Province, China). The dosages of anesthetics for induction and maintenance were selected based on standard clinical practices at our institution and widely accepted guidelines for thoracic surgery, aiming to ensure adequate depth of anesthesia, hemodynamic stability, and optimal surgical conditions. During anesthesia maintenance, a fresh air-oxygen mixture (oxygen concentration of 50%-80%, flow rate of 2 L/min) combined with remifentanil (Approval No. H20030197, National Medical Products Administration) was used at an infusion rate of 0.05-0.20 μg/(kg·min), maintaining sevoflurane at 0.7-1.3 MAC.

After general anesthesia intubation and before the start of surgery, both groups received an ultrasound-guided ESPB on the affected side administered by the same experienced anesthesiologist. Patients were placed in a lateral decubitus position with the operative side up. The skin was routinely disinfected and draped, and a high-frequency linear ultrasound probe was positioned longitudinally in the sagittal plane to locate the T5 spinous process. The probe was then moved laterally by 3 cm to identify the T5 transverse process. Under in-plane needle insertion, the needle was advanced until it contacted the bony structure of the T5 transverse process. After confirming no blood or cerebrospinal fluid upon aspiration, the prepared local anesthetic was slowly injected. For the liposomal bupivacaine group, liposomal bupivacaine 266 mg (20 mL) (Approval No. H20223899, Jiangsu Hengrui Medicine Co., Ltd., Jiangsu Province, China) was diluted with 10 mL of normal saline to a total volume of 30 mL. This dose (266 mg) represents the standard single vial presentation of liposomal bupivacaine and is within the recommended maximum for regional anesthesia. Dilution to 30 mL aimed to ensure adequate spread within the erector spinae plane to cover the T3-T7 segments. For the bupivacaine group, bupivacaine 20mL (100 mg) (Approval No. H43021409, Hunan Kelun Pharmaceutical Co., Ltd., Hunan Province, China) was diluted with normal saline to a total volume of 30 mL. The 100 mg dose of bupivacaine was chosen as it is a commonly reported and effective dose for ESPB, and the total volume was matched between groups to standardize the block technique. After injection, the spread of the local anesthetic between the erector spinae muscle and the costotransverse ligament, forming a “hypoechoic area” on the ultrasound image, was observed to ensure that the drug diffusion covered the T3-T7 segments.

All surgeries were performed by the same thoracic surgeon using VATS technology. An 8 mm incision was made at the seventh or eighth intercostal space along the posterior axillary line for the camera, and a 4 cm incision was made at the first or fifth intercostal space along the anterior axillary line for surgical procedures. The intrathoracic structures were observed through the thoracoscope, and standard thoracoscopic instruments were used to perform a radical lung cancer resection (lobectomy, segmentectomy, or wedge resection depending on the tumor location and size). During the surgery, the mean arterial pressure was maintained within 20% of the baseline value, and the BIS of the electroencephalogram was kept between 40 and 60.

### 2.3 Postoperative management

After surgery, patients were transferred to the Post-Anesthesia Care Unit (PACU) and received patient-controlled intravenous analgesia (PCIA) upon regaining consciousness. The PCIA regimen consisted of sufentanil 2.50 μg/kg (maximum dose 200.00 μg) diluted with normal saline to a total volume of 150 mL. The parameters were set as follows: bolus dose of 1.5 mL, lockout interval of 5 minutes, no background infusion, and a maximum hourly limit of 6 mL, with the catheter left in place for 72 hours. Pain intensity was assessed using the Visual Analog Scale (VAS). If the VAS score at rest was ≥4 despite the PCIA, a loading dose of 4 mL of sufentanil was administered, and the maximum hourly limit was temporarily increased to 8 mL. This protocol allowed for rapid rescue analgesia while maintaining a safety ceiling to prevent over-sedation. Patients were closely monitored for any adverse effects following these interventions.

The PCIA was the primary analgesic modality in this study. Intravenous non-steroidal anti-inflammatory drugs (NSAIDs) were not routinely administered during the study period due to institutional protocols at that time that emphasized cautious use in lung surgery patients over concerns for potential effects on platelet function and renal perfusion, although they are recognized components of modern ERAS bundles. Regular scheduled acetaminophen (paracetamol) was not part of the standard intravenous order set but could be provided orally upon patient request after resuming oral intake, which typically occurred on postoperative day 1. This background analgesic regimen was consistent across both study groups.

### 2.4 Outcome assessment

The primary outcomes of this study were postoperative pain levels and early functional recovery scores. Pain levels were assessed using the Visual Analog Scale (VAS) at the Post-Anesthesia Care Unit (PACU) and at 24, 48, and 72 hours postoperatively. The VAS scoring ranged from 0 cm (no pain) to 10 cm (most severe pain), including both at rest and during coughing. The intraclass correlation coefficient (ICC) for this scale was 0.97 (15). Overall functional recovery was evaluated using the total score of the 15-item Quality of Recovery (QoR-15) at 24, 48, and 72 hours postoperatively. The scoring range for the QoR-15 was from 0 to 150, with higher scores indicating better functional recovery. The Cronbach’s α coefficient for this scale was 0.76 (16). The QoR-15 questionnaire was integrated into our standard postoperative nursing assessment protocol during the study period. Nurses administered the questionnaire at the specified time points, and scores were documented in the electronic medical record, allowing for reliable retrospective extraction.

Secondary outcomes included postoperative opioid consumption (0–24 hours, 24–48 hours, and 48–72 hours), pulmonary function indices (forced expiratory volume in the first second [FEV1] and forced vital capacity [FVC] at 24, 48, and 72 hours postoperatively), perioperative adverse events (including dizziness, postoperative nausea and vomiting [PONV], pruritus, hypotension, and bradycardia), and other postoperative-related outcomes (including time to first ambulation, time to first request for analgesia, chest tube removal time, length of hospital stay, and the incidence of chronic post-surgical pain [CPSP] at three months postoperatively). Pulmonary function indices were measured using a portable spirometer (H1-101, CHEST M.I., Inc., Japan).

### 2.5 Statistical analysis

In this study, the statistical analyses were performed utilizing SPSS statistical software (version 29.0; developed by SPSS Inc., Chicago, IL, USA). Following validation with the Shapiro-Wilk test, it was established that every continuous variable analyzed adhered to a normal distribution, hence they are reported as mean ± standard deviation (M ± SD). To compare groups, independent samples t-tests were employed. Categorical variables are shown as counts and proportions [n (%)] and inter-group comparisons were conducted using the chi-square (χ²) test. Statistical significance was set at a P value of less than 0.05.

Given the exploratory and comparative nature of this retrospective study, which aimed to describe potential differences across a broad set of recovery metrics, adjustments for multiple comparisons were not performed. The p-values are presented descriptively, and emphasis is placed on the consistency of findings across related clinical domains.

## 3 Results

### 3.1 General data

The comparison of demographic characteristics between the Bupivacaine group and the Liposomal bupivacaine group (**Table 1**) showed no significant differences. Age (P = 0.472), gender distribution (P = 0.358), BMI (P = 0.620), ethnicity (P = 0.538), educational level (P = 0.809), marital status (P = 0.633), smoking history (P = 0.527), drinking history (P = 0.597), hypertension prevalence (P = 0.815), diabetes prevalence (P = 0.457), and ASA physical status (P = 0.703) were all similar between the two groups.

**Table 1:** Comparison of demographic characteristics between two groups.

Parameters	Bupivacaine group (n=115)	Liposomal bupivacaine group (n=131)	t/χ^2^	P
Age (years)	55.42 ± 7.48	56.13 ± 7.94	0.720	0.472
Gender [n(%)]			0.845	0.358
Male	60 (52.17%)	76 (58.02%)		
Female	55 (47.83%)	55 (41.98%)		
BMI (kg/m^2^)	22.65 ± 1.24	22.73 ± 1.31	0.496	0.620
Ethnicity [n(%)]			0.379	0.538
Han	110 (95.65%)	123 (93.89%)		
Others	5 (4.35%)	8 (6.11%)		
Educational level [n(%)]			0.059	0.809
Junior college graduate or lower	97 (84.35%)	109 (83.21%)		
College graduate or higher	18 (15.65%)	22 (16.79%)		
Marital status [n(%)]			0.228	0.633
Married	108 (93.91%)	121 (92.37%)		
Unmarried	7 (6.09%)	10 (7.63%)		
Smoking history [n(%)]			0.400	0.527
Yes	42 (36.52%)	53 (40.46%)		
No	73 (63.48%)	78 (59.54%)		
Drinking history [n(%)]			0.279	0.597
Yes	35 (30.43%)	44 (33.59%)		
No	80 (69.57%)	87 (66.41%)		
Underlying disease				
Hypertension [n(%)]	26 (22.61%)	28 (21.37%)	0.054	0.815
Diabetes [n(%)]	13 (11.30%)	19 (14.50%)	0.554	0.457
ASA physical status [n(%)]			0.146	0.703
I	36 (31.30%)	44 (33.59%)		
II	79 (68.70%)	87 (66.41%)		

BMI, Body Mass Index; ASA, American Society of Anesthesiologists.

The comparison of surgical features between the Bupivacaine group and the Liposomal bupivacaine group (**Table 2**) showed no significant differences. Surgery type distribution (P = 0.923), duration of surgery (P = 0.643), intraoperative bleeding volume (P = 0.109), intraoperative sufentanil dosage (P = 0.328), and intraoperative remifentanil dosage (P = 0.617) were all similar between the two groups.

**Table 2:** Comparison of surgical features between two groups.

Parameters	Bupivacaine group (n=115)	Liposomal bupivacaine group (n=131)	t/χ^2^	P
Surgery type [n(%)]			0.161	0.923
Lobectomy	48 (41.74%)	58 (44.27%)		
Segmentectomy	32 (27.83%)	35 (26.72%)		
Wedge resection	35 (30.43%)	38 (29.01%)		
Duration of surgery (min)	125.43 ± 30.54	127.29 ± 31.94	0.464	0.643
Intraoperative bleeding volume (mL)	147.05 ± 28.71	152.62 ± 25.63	1.606	0.109
Intraoperative sufentanil dosage (μg)	35.09 ± 8.92	36.25 ± 9.57	0.980	0.328
Intraoperative remifentanil dosage (mg)	1.34 ± 0.36	1.36 ± 0.41	0.501	0.617

### 3.2 Postoperative pain levels

The comparison of VAS scores between the Bupivacaine group and the Liposomal bupivacaine group (**Table 3**) showed significant differences at rest. In the PACU, VAS scores were lower in the Liposomal bupivacaine group (3.95 ± 0.28 vs 4.09 ± 0.42, t=3.088, P = 0.002). At postoperative 24 hours, the Liposomal bupivacaine group had lower scores (4.73 ± 0.49 vs 4.94 ± 0.65, t=2.901, P = 0.004), and this trend continued at postoperative 48 hours (2.46 ± 0.27 vs 2.58 ± 0.48, t=2.274, P = 0.024). These statistically significant differences, though numerically modest, are considered clinically relevant as they represent a consistent and meaningful reduction in pain during the critical early recovery phase, potentially facilitating deeper breathing, coughing, and initial mobilization. No significant difference was observed at postoperative 72 hours (P = 0.133). The lack of a significant difference at 72 hours likely reflects the resolution of acute surgical pain in both groups and the diminished effect of the single-injection blocks, suggesting that the primary analgesic benefit of liposomal bupivacaine lies in enhancing early recovery. For VAS scores during coughing, no significant differences were found at any time points: PACU (P = 0.745), postoperative 24 hours (P = 0.145), postoperative 48 hours (P = 0.889), and postoperative 72 hours (P = 0.966).

**Table 3:** Comparison of VAS between two groups (scores).

Parameters	Bupivacaine group (n=115)	Liposomal bupivacaine group (n=131)	t	P
At rest				
PACU	4.09 ± 0.42	3.95 ± 0.28	3.088	0.002
Postoperative 24h	4.94 ± 0.65	4.73 ± 0.49	2.901	0.004
Postoperative 48h	2.58 ± 0.48	2.46 ± 0.27	2.274	0.024
Postoperative 72h	0.94 ± 0.26	0.89 ± 0.22	1.506	0.133
At cough				
PACU	4.07 ± 0.43	4.05 ± 0.41	0.325	0.745
Postoperative 24h	7.76 ± 0.46	7.68 ± 0.44	1.462	0.145
Postoperative 48h	5.44 ± 0.39	5.43 ± 0.36	0.140	0.889
Postoperative 72h	3.32 ± 0.37	3.32 ± 0.35	0.043	0.966

VAS, Visual Analogue Scale; PACU, Post Anesthesia Care Unit.

### 3.3 Early functional recovery

**Figure 2:**
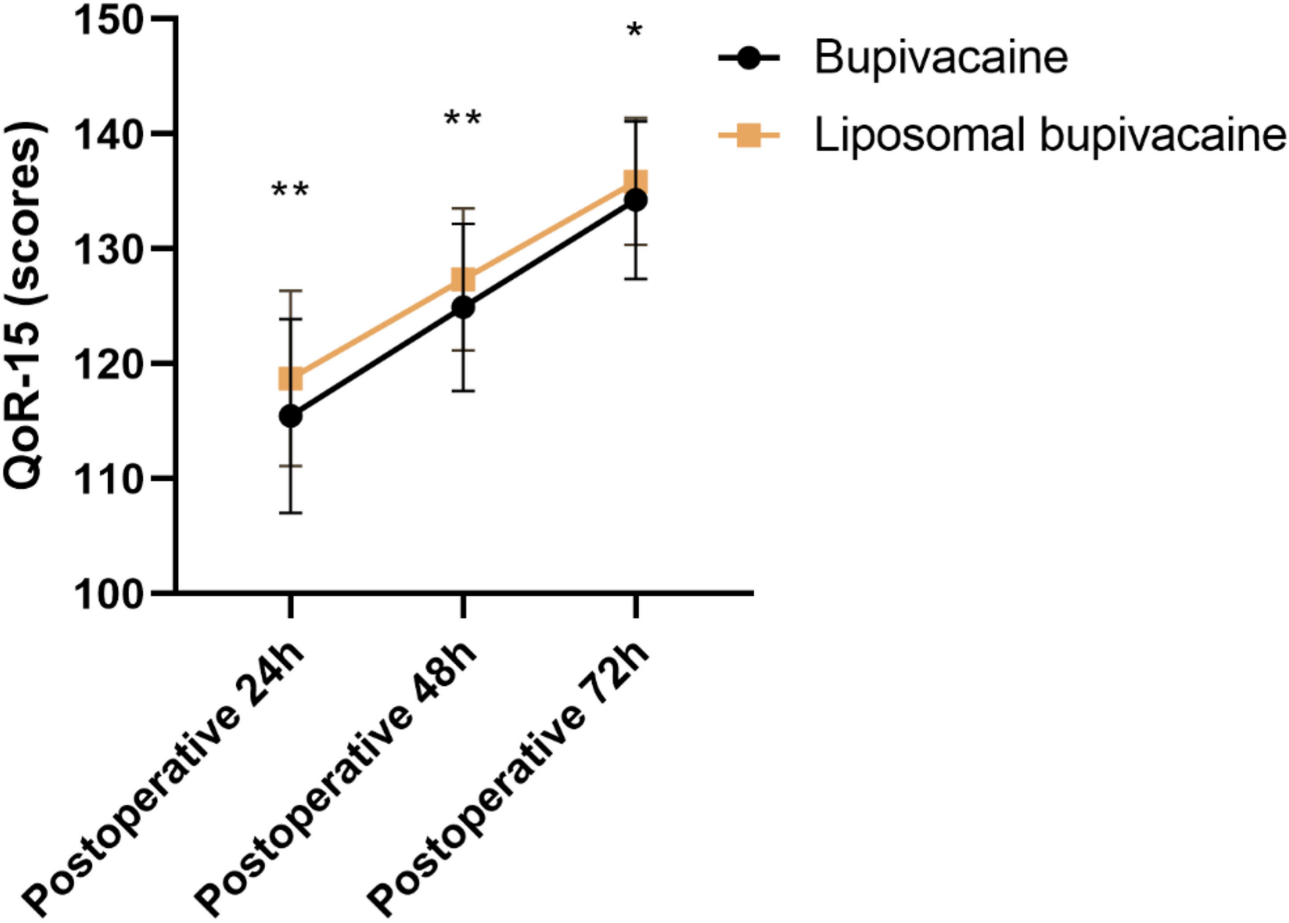
Comparison of QoR-15 between two groups. (scores). QoR-15, Quality of Recovery-15; *P<0.05 Bupivacaine group compared with Liposomal bupivacaine group; **P<0.01 Bupivacaine group compared with Liposomal bupivacaine group.

The comparison of QoR-15 scores between the Bupivacaine group and the Liposomal bupivacaine group (**Figure 2**) showed significant differences. At postoperative 24 hours, the Liposomal bupivacaine group had higher scores (118.74 ± 7.63 vs 115.46 ± 8.45, t=3.198, P = 0.002). This trend persisted at postoperative 48 hours (127.35 ± 6.17 vs 124.92 ± 7.28, t=2.833, P = 0.005) and postoperative 72 hours (135.86 ± 5.52 vs 134.25 ± 6.84, t=2.019, P = 0.045). The higher QoR-15 scores in the liposomal bupivacaine group, which assesses domains such as physical comfort, independence, and psychological support, correlate with the observed clinical milestones of earlier ambulation and reduced opioid-related side effects, indicating a more robust and comprehensive recovery profile.

### 3.4 Postoperative opioid consumption

**Figure 3:**
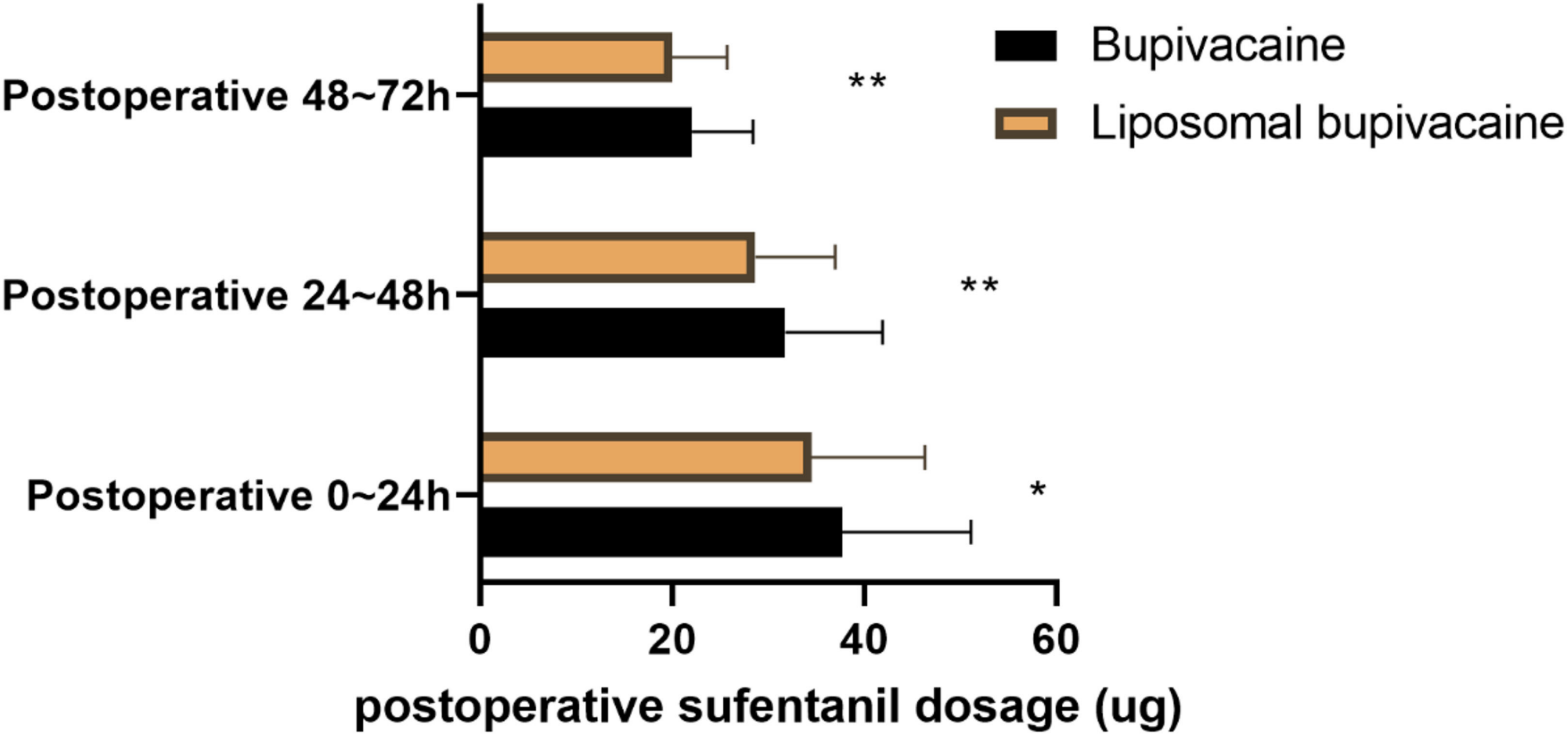
Comparison of postoperative sufentanil dosage between two groups. (ug). *P<0.05 Bupivacaine group compared with Liposomal bupivacaine group; **P<0.01 Bupivacaine group compared with Liposomal bupivacaine group.

The comparison of postoperative sufentanil dosage between the Bupivacaine group and the Liposomal bupivacaine group (**Figure 3**) showed significant differences at all time points. During the first 24 hours postoperatively, the Liposomal bupivacaine group required less sufentanil (34.51 ± 11.84 vs 37.73 ± 13.42, t=1.999, P = 0.047). From 24 to 48 hours postoperatively, this difference persisted (28.67 ± 8.32 vs 31.79 ± 10.15, t=2.619, P = 0.009). Similarly, from 48 to 72 hours postoperatively, the Liposomal bupivacaine group used less sufentanil (19.98 ± 5.75 vs 22.04 ± 6.37, t=2.669, P = 0.008). Minimizing opioid exposure in the acute postoperative period may lower the risk of persistent opioid use and the development of opioid-induced hyperalgesia, thereby contributing to more favorable chronic pain outcomes.

### 3.5 Pulmonary function indices

The comparison of pulmonary function indices between the Bupivacaine group and the Liposomal bupivacaine group (**Table 4**) showed significant differences. For FEV1, the Liposomal bupivacaine group had higher values at postoperative 24 hours (1.68 ± 0.31 vs 1.59 ± 0.35, t=2.210, P = 0.028), postoperative 48 hours (1.95 ± 0.29 vs 1.86 ± 0.33, t=2.208, P = 0.028), and postoperative 72 hours (2.16 ± 0.27 vs 2.07 ± 0.31, t=2.457, P = 0.015).

**Table 4:** Comparison of pulmonary function indices between two groups (L).

Parameters	Bupivacaine group (n=115)	Liposomal bupivacaine group (n=131)	t	P
FEV_1_				
Postoperative 24h	1.59 ± 0.35	1.68 ± 0.31	2.210	0.028
Postoperative 48h	1.86 ± 0.33	1.95 ± 0.29	2.208	0.028
Postoperative 72h	2.07 ± 0.31	2.16 ± 0.27	2.457	0.015
FVC				
Postoperative 24h	2.06 ± 0.42	2.18 ± 0.38	2.393	0.017
Postoperative 48h	2.39 ± 0.39	2.49 ± 0.35	2.123	0.035
Postoperative 72h	2.68 ± 0.36	2.76 ± 0.32	1.775	0.077

FEV_1_, Forced Expiratory Volume in the First Second; FVC, Forced Vital Capacity.

For FVC, the Liposomal bupivacaine group also demonstrated higher values at postoperative 24 hours (2.18 ± 0.38 vs 2.06 ± 0.42, t=2.393, P = 0.017) and postoperative 48 hours (2.49 ± 0.35 vs 2.39 ± 0.39, t=2.123, P = 0.035). No significant difference was observed at postoperative 72 hours (P = 0.077).

### 3.6 Perioperative adverse events

The comparison of perioperative adverse events between the Bupivacaine group and the Liposomal bupivacaine group (**Table 5**) revealed no significant differences. Dizziness (P = 0.154), PONV (P = 0.965), pruritus (P = 0.565), hypotension (P = 0.134), and bradycardia (P = 0.248) were all similar between the two groups.

**Table 5:** Comparison of perioperative adverse events between two groups [n(%)].

Parameters	Bupivacaine group (n=115)	Liposomal bupivacaine group (n=131)	χ^2^	P
Dizziness	14 (12.17%)	9 (6.87%)	2.032	0.154
PONV	6 (5.22%)	7 (5.34%)	0.002	0.965
Pruritus	4 (3.48%)	2 (1.53%)	0.332	0.565
Hypotension	22 (19.13%)	16 (12.21%)	2.243	0.134
Bradycardia	18 (15.65%)	14 (10.69%)	1.334	0.248

PONV, Postoperative Nausea and Vomiting.

### 3.7 Other postoperative-related outcomes

The comparison of other postoperative-related outcomes between the Bupivacaine group and the Liposomal bupivacaine group (**Table 6**) showed significant differences in certain parameters. The time to first ambulation was shorter in the Liposomal bupivacaine group (16.83 ± 5.27 vs 18.45 ± 6.24, t=2.201, P = 0.029), and the time to first request for analgesia was longer (584.18 ± 121.56 vs 514.35 ± 100.97, t=4.92, P<0.001). No significant differences were found in chest tube removal time (P = 0.077), lengths of hospital stay (P = 0.386), or CPSP at three months after surgery (P = 0.750). The lack of significant difference in CPSP at three months may be influenced by the multifactorial nature of chronic pain, including surgical technique, pre-existing pain conditions, and psychosocial factors, which a single perioperative intervention may not fully override. Similarly, the absence of a significant reduction in hospital stay or chest tube duration suggests that these outcomes are likely governed by a broader set of clinical protocols and patient factors beyond the scope of analgesia provided by the nerve block, highlighting that while liposomal bupivacaine ESPB improves key recovery metrics, it is one component within a multifaceted postoperative care pathway.

**Table 6:** Comparison of other postoperative-related outcomes between two groups.

Parameters	Bupivacaine group (n=115)	Liposomal bupivacaine group (n=131)	t/χ^2^	P
Time to first ambulation (h)	18.45 ± 6.24	16.83 ± 5.27	2.201	0.029
Time to first request for analgesia (min)	514.35 ± 100.97	584.18 ± 121.56	4.92	<0.001
Chest tube removal time (d)	3.28 ± 1.05	3.04 ± 1.01	1.774	0.077
Lengths of hospital stay (d)	9.46 ± 3.09	9.13 ± 2.84	0.869	0.386
CPSP (3 months after surgery) [n(%)]	22 (19.13%)	23 (17.56%)	0.101	0.750

CPSP, Chronic Postsurgical pain.

## 4 Discussion

The present study aimed to evaluate the impact of liposomal bupivacaine ESPB on postoperative analgesia and early functional recovery in patients undergoing thoracoscopic lung cancer surgery. Our findings suggest that liposomal bupivacaine ESPB offers several advantages over conventional bupivacaine. These benefits are likely mediated through multiple mechanisms, including prolonged local anesthetic action, reduced systemic absorption, and improved patient comfort.

The observed superior analgesic profile associated with liposomal bupivacaine may be explained by its unique formulation. The encapsulation of bupivacaine within multivesicular liposomes allows for a slow, sustained release at the site of injection (9, 17). This prolonged release maintains more stable plasma concentrations and provides continuous neural blockade, which could contribute to the sustained pain relief we observed (18–20). Consequently, patients receiving liposomal bupivacaine reported lower VAS scores at rest during the first 48 hours postoperatively. The lack of a significant difference in cough-induced VAS scores, despite the improvement at rest, may be due to the nature of cough as an acute, high-intensity stimulus that can transiently overwhelm a regional block, making both groups similarly reliant on the available supplemental opioid rescue. This consistent analgesia is essential in the early phase, as it enables patients to perform essential recovery activities, such as deep breathing and coughing, with less discomfort.

The observed differences in resting VAS scores, while statistically significant, were numerically modest. It is therefore important to consider their clinical relevance. In the acute postoperative setting, even small reductions in pain can be meaningful if they enable critical recovery behaviors. A decrease of 1 point or 10-20% on the VAS is often considered a minimal clinically important difference in studies of acute pain. Our findings approach or meet this threshold at several time points. The consistency of lower pain scores across the first 48 hours, combined with the concurrently observed significant reductions in opioid consumption and improvements in functional recovery metrics, suggests that the aggregate clinical benefit may be substantive. This multimodal advantage is a central finding of our study. We interpret these results cautiously, recognizing that the primary advantage of liposomal bupivacaine in this context may lie in its sustained effect profile and opioid-sparing potential, which collectively support enhanced recovery, rather than in dramatically lower peak pain scores.

Enhanced early functional recovery is another important outcome in postoperative care, as it contributes to faster hospital discharge and better long-term outcomes. Patients in the liposomal bupivacaine group achieved higher QoR-15 scores at various time points postoperatively compared to those in the conventional bupivacaine group. This validated patient-reported outcome measure captures critical aspects of recovery beyond pain, including physical independence, psychological well-being, and overall patient satisfaction. Our results demonstrate that improved analgesia directly contributes to a better perceived recovery experience. The superior pain control likely enabled patients to engage more actively in rehabilitation (12, 21, 22).

Postoperative spirometry at 24, 48, and 72 hours is a routine component of our institutional enhanced recovery pathway for thoracic surgery patients. These measurements provide objective data to guide respiratory therapy, assess readiness for chest tube removal, and identify patients at risk for pulmonary complications. Although the absolute differences in FEV1 and FVC between groups were modest, they were consistent across time points and statistically significant. Even small improvements in early postoperative pulmonary function can facilitate more effective coughing, deeper breathing, and earlier mobilization, which are clinically meaningful in reducing atelectasis and pneumonia risk. The additional cost of spirometry is minimal, as it is performed using portable devices during routine nursing assessments and does not entail extra laboratory fees or extended hospitalization.

Opioid-sparing effects are crucial for minimizing the adverse effects associated with opioid use, such as nausea, vomiting, dizziness, and respiratory depression. Our results demonstrate that patients in the liposomal bupivacaine group required less postoperative sufentanil compared to those in the conventional bupivacaine group. The sustained release mechanism of liposomal bupivacaine ensures continuous pain relief, thereby reducing the need for supplemental opioids. This reduction in opioid consumption not only mitigates the risk of opioid-related complications but also enhances patient satisfaction and comfort (23). Furthermore, minimizing opioid use can help prevent the development of opioid dependence, a growing concern in modern healthcare settings (24, 25).

Thoracoscopic lung cancer surgery can significantly affect pulmonary function due to the surgical trauma and subsequent inflammation. Patients receiving liposomal bupivacaine ESPB exhibited better pulmonary function indices, including FEV1 and FVC, compared to those receiving conventional bupivacaine. Better pulmonary function indices were associated with liposomal bupivacaine ESPB. This may be related to more effective pain control, potentially enabling patients to breathe more deeply and cough more effectively without experiencing severe pain. Effective coughing and deep breathing are essential for clearing secretions and preventing atelectasis and other pulmonary complications. In addition, reduced opioid consumption in the liposomal bupivacaine group may contribute to better pulmonary function by avoiding opioid-induced respiratory depression and sedation (7, 26, 27).

Early ambulation is a key component of ERAS protocols, as it promotes circulation, reduces the risk of thromboembolic events, and accelerates overall recovery. Patients in the liposomal bupivacaine group ambulated earlier than those in the conventional bupivacaine group. This earlier ambulation can be attributed to the improved pain control and reduced opioid consumption observed in the liposomal bupivacaine group. Similarly, the time to the first request for analgesia was longer in the liposomal bupivacaine group, indicating prolonged pain relief and reduced demand for additional analgesics (12, 28).

While no significant differences were observed in perioperative adverse events between the two groups, it is worth noting that both groups experienced low rates of adverse events such as dizziness, postoperative nausea and vomiting (PONV), pruritus, hypotension, and bradycardia. This suggests that both bupivacaine and liposomal bupivacaine ESPB are safe and well-tolerated in this patient population (22, 29, 30). The absence of significant differences in adverse events further supports the safety profile of liposomal bupivacaine, reinforcing its potential as a viable alternative to conventional bupivacaine for postoperative pain management.

Our findings align with recent studies supporting the use of liposomal bupivacaine in regional anesthesia for thoracic surgery. For example, a randomized controlled trial by Chi et al. (31) demonstrated that liposomal bupivacaine provided superior and sustained analgesia with reduced opioid demand compared to conventional bupivacaine in thoracoscopic surgery patients. These results are consistent with our observations, reinforcing the clinical value of liposomal bupivacaine in enhancing postoperative recovery pathways.

The clinical implications of our findings are substantial. Integrating liposomal bupivacaine ESPB into standardized pain management protocols for VATS could optimize recovery pathways. This technique facilitates key ERAS milestones, such as early ambulation and improved pulmonary function, potentially leading to more predictable and efficient patient recovery. The technique’s promise may extend to other surgical domains where prolonged regional analgesia is desired. Our study also highlights the importance of focusing on patient-centric outcomes. The higher QoR-15 scores in the liposomal bupivacaine group reflect a recovery quality that patients value. Moving beyond traditional metrics like pain scores alone, to encompass overall recovery quality and patient satisfaction, is essential for evaluating the true success of perioperative care strategies.

Despite the promising findings, our study has several limitations that warrant consideration. Being a retrospective analysis, there is a potential for selection bias and confounding variables that may have influenced the results. Although our analysis showed balanced baseline characteristics between groups, we acknowledge that residual confounding from unmeasured or imperfectly measured factors (e.g., subtle differences in surgical dissection, individual patient pain tolerance, or psychosocial variables) cannot be excluded. While a multivariable regression could adjust for known covariates, in a retrospective study it cannot fully account for hidden biases inherent in treatment allocation. Therefore, the associations we report, while adjusted for in design by temporal grouping and analyzed with statistical tests for group differences, should be interpreted as generating hypotheses rather than confirming definitive effects. Future prospective randomized controlled trials (RCTs) are needed to validate these findings and establish causality. The non-randomized, time-based allocation of patients to the two bupivacaine formulations may introduce temporal confounding, such as subtle changes in surgical technique, nursing care, or perioperative protocols over the study period. Although we controlled for key demographic and surgical variables, unmeasured confounders could influence outcomes. Future prospective randomized trials are warranted to eliminate such biases and establish causal efficacy. While we evaluated various parameters related to postoperative pain management and functional recovery, we did not assess long-term outcomes such as chronic post-surgical pain (CPSP). Longitudinal studies focusing on the long-term benefits of liposomal bupivacaine ESPB would provide valuable insights into its sustained efficacy. In addition, it was conducted at a single center, which may limit generalizability. The cost-effectiveness of liposomal bupivacaine compared to conventional bupivacaine, especially when considering the potential savings from reduced opioid use and faster recovery, remains an important area for future health-economic analyses.

Our study suggests that liposomal bupivacaine ESPB offers several advantages over conventional bupivacaine in managing postoperative pain, reducing opioid consumption, improving pulmonary function, and promoting early functional recovery in patients undergoing thoracoscopic lung cancer surgery. These benefits are likely mediated through mechanisms such as prolonged local anesthetic action, reduced systemic absorption, and enhanced patient comfort. While further research is needed to confirm these findings and explore their long-term implications, the use of liposomal bupivacaine ESPB represents a promising approach for optimizing postoperative care in this patient population. Future studies should focus on validating these results in larger, more diverse populations and assessing the long-term outcomes and cost-effectiveness of this technique.

## 5 Conclusion

In this retrospective analysis, the use of liposomal bupivacaine for ESPB was associated with significantly better postoperative analgesia and early functional recovery in patients undergoing thoracoscopic lung cancer surgery. Patients receiving liposomal bupivacaine reported lower pain scores and reduced opioid consumption, alongside higher QoR-15 scores and shorter times to first ambulation. The sustained release of bupivacaine from its liposomal formulation minimizes systemic absorption, enhancing patient comfort and reducing the risk of opioid-related side effects. Future research should focus on its long-term efficacy, safety, and cost-effectiveness across diverse patient populations.

## Data Availability

The raw data supporting the conclusions of this article will be made available by the authors, without undue reservation.
